# Bibliographical Notices

**Published:** 1854-07

**Authors:** 


### BIBLIOGRAPHICAL NOTICES.

*On Rheumatism, Rheumatic Gout, and Sciatica; their Pathology, Symptoms and Treatment.* By Henry William Fuller, M. D., Cantab. (Reprint.) N. York, S. S. & W. Wood; 8vo., pp. 322.

This work recommends itself even to the superficial reader, by its scholarly tone and its comprehensive scope, but its thoroughness and precision render it equally acceptable to the scientific enquirer and the practitioner of medicine. That it is chargeable with certain defects of arrangement, omissions of fact and errors of reasoning we believe, yet we are also of opinion that it contains a completer description of rheumatism in its manifold phases and relations than is to be met with in any other treatise upon the subject. The narrow limits of this notice preclude the possibility of examining the work in detail; a consideration, therefore, of some of its more striking features is all that will be attempted.

The first and second chapters treat of the causes of rheumatism; the third of its seat and varieties; the fourth and fifth of the acute form and its treatment; the sixth, seventh and eighth of rheumatic affections of the heart and their treatment; the ninth of the statistics bearing upon the last named subjects; the tenth of rheumatic cerebral and pulmonary affections, and disorganization of the joints; the eleventh of rheumatic gout; the twelfth of chronic rheumatism; and the thirteenth of sciatica and other forms of neuralgic rheumatism. This arrangement, it is evident, is very comprehensive, the author has rendered it also very full. The subdivisions of the chapters are not always equally logical, and were it not for an analytical table of contents, the labor of referring to particular subjects would be likely to deter many who might, nevertheless, find the result a profitable one. In all works intended for study and reference there should be running titles, marginal analyses, headings to paragraphs, &c. If these helps were thought essential in days when volumes were few in number and apt to be thoroughly studied, they are still more so now when the multiplication of books is infinite, and so many aspire to a permanent place in our professional literature.

In the Preface we are assured that the work has been composed with a constant reference to facts, and, upon the whole, there is good evidence that it has been so. In the first chapter, however, there is a notable example of the contrary. Cold, as every one knows, has been in all ages regarded as the chief cause of rheumatism, but, says our author, "though the ascertained local and general effects of cold differ altogether from the symptoms of rheumatism, yet some, even among the profession, have been found to adopt this prevalent notion, without an attempt to show that cold either does or can produce the effects assigned to it." He then proceeds to illustrate the effects of cold by describing the primary action of it upon the skin, &c., and its secondary consequences, frost-bite, gangrene, &c. Now, as every body knows who has seen or suffered lumbago, stiff-neck, or pain and stiffness of a joint from a draught of air—and who has not?—cold produces other effects besides those just mentioned, produces in fact rheumatism. That the abstraction of heat is *all* that intervenes between the action of the cause and the symptoms of the disease, as seems to be more nearly the case in frost-bite, no one will maintain, and no one did ever assert; but because that intermediate link in the chain of effects is unknown, we must, as in all similar cases, assume the remoter for the proximate cause, and still continue to say that the chief and probably the only external cause of rheumatism is cold. Dr. Fuller, indeed, after denying the agency of cold in the passage above quoted, as well as in others, and laboring to show how very different the effects of cold are from the phenomena of rheumatism, admits (p. 37) that cold and other external agencies are predisposing and exciting causes of the disease. But not content with this conclusion, which is *urbi et orbi recepta,* he contends for the real existence of a proximate cause, and, like various other writers, finds it in "the presence of a morbid matter in the blood, generated in the system as the product of a peculiar form of mal-assimilation—of vicious metamorphic action. This poison it is which excites the fever and produces all the pains and local inflammations," &c. (ibid.) Of the nature of the supposed poison he says, (p. 31,) "It is probable, therefore, as the skin is the peculiar emunctory of lactic acid, that in it we have discovered the actual materies morbi." This hypothesis was first proposed, we believe, by the ingenious Dr. Prout, but although many years have elapsed since then, it still remains in the chrysalis state of a hypothetical postulate, and even the assistance of our author has not enabled it to stand self-poised. Until its completer development takes place we may be satisfied in assigning cold as the chief cause of rheumatism, while we describe the retention in the blood of the normal excretion from the skin as one of the ordinary phenomena of the disease.

Having thus endeavored to state the relations to one another of the causes upon which rheumatism depends, several subordinate sources of the disease may be alluded to; those mentioned by the author are indigestion, defective or perverted uterine action, ill performance of the renal functions, excessive venery and debauchery, prolonged lactation, and an inherited rheumatic taint. In all of these, except the last, there is the common element of debility, proneness to suffer from changes of temperature, susceptibility to cold. Cold, after all, then, would seem to be the unique cause of the accidental disease. Not that the temperature of the air need be very low—for the feeble and susceptible are chilled, and experience all the phenomena of coldness, when the robust and healthy are oppressed with heat—but only that it be such as to produce the sensation of cold, with arrest of the perspiration, &c.

According to Dr. Fuller, "rheumatism, like gout, is distinctly hereditary." The grounds of this statement are the results of investigations among *hospital patients.* As well might one say that poverty is hereditary. Upon the data furnished by the same class of patients, the latter proposition would undoubtedly be found every whit as true as the former. Gout, it is well known, attacks the children, the grandchildren even, of those whose debauchery has occasioned the disease in themselves, although their descendants have led entirely temperate lives. There is no evidence that the same can be said of rheumatism. The poor, and all whose occupations expose them habitually to vicissitudes of weather, are the chief subjects of rheumatism. Their children, leading the same sort of life, ill clad, ill fed, ill lodged, laboring in tempest and calm, in winter and summer, robbed of their natural rest, and perhaps exhausted by dissipation, may reckon causes enough of rheumatism in these hardships, without having to reproach their parents with an additional inheritance of suffering.

The author says (p. 49) "that no one texture is exclusively the seat of irritation in rheumatism," . . . "for, if rheumatic inflammation be due to the presence of an irritating matter in the blood, it is obvious that *all* must be more or less liable to suffer." He then goes on to show that "the textures most commonly implicated in rheumatism are all examples of the albuminous and gelatinous tissues," the fibrous tissues, as they are commonly called. In other words, his second proposition contradicts his first. Still, we would only take exception to his inference that *all* parts are more or less liable to suffer. On the contrary, if the symptoms of rheumatism be due to lactic acid or other material substance in the blood, it would be most apt to accumulate in the white tissues where the blood vessels are smallest; and these are precisely the tissues most affected. The explanation is more consistent than the author's; but both are merely speculative. It is of more consequence and interest to know that the joints most exercised are those which are most apt to be attacked, and that corresponding joints on opposite sides of the body are most frequently the seats of the disease, the latter fact showing the diffusion of the material cause of the disease through the system, and the former pointing to the mode in which the occasional cause—cold—acts by arresting perspiration where it is most profuse, and where the exhaustion of labor renders the part least able to resist and overcome the morbid impression.

The symptomatology of acute rheumatism, described in Chapter IV., is very minute, complete and clear, and is probably as near the truth as any general description can be. The author regards as erroneous the opinion that the profuse perspiration of the disease is enfeebling and wasting, and neither alleviates the patients' sufferings nor shortens their duration. He grounds his belief upon the fact that if they are checked for a time the symptoms grow worse, and he states that the more profuse and acid the sweats are, the shorter is the duration of the attack, *provided* they do not occur in debilitated and cachectic states of the constitution. With this proviso his opinion is perhaps correct. Certain it is, that those who are much weakened by the sweats are not benefitted by them, nor do they emit the characteristic acid smell of such as tend to the resolution of the disease.

As regards the treatment of acute rheumatism, the author condemns, unequivocally and very properly, the copious and repeated blood-letting practiced by certain modern Sangrados. He believes that it retards convalescence and favors relapse, and therefore does not recommend depletion as a part of ordinary practice, but as suitable only for robust and plethoric individuals in whom the febrile reaction runs high. Active purging he condemns no less strongly, and prescribes aperients only when palpable derangement of the chylopoietic viscera or decided constipation indicate their propriety. Of opium he speaks in terms of merited approbation, and confirms the assertion of all who have used this medicine freely, that very large doses of it are not only borne without inconvenience, but that they promote, in a remarkable degree, the patient's comfort, while they often hasten the period of convalescence, and lessen the frequency of inflammation of the heart. He recommends from six to twelve grains to be given in the twenty-four hours, but we think that where many joints are attacked, when the pain is severe and the patient irritable, much larger doses even than these may be prescribed with advantage. Sweating by hot vapor, &c., the author condemns; but if his hypothesis of the nature of the disease were true, this remedy ought to be the sheet-anchor of his mode of treatment. Fortunately, he dismounts from his hobby when experience arrests its motions. Mercurials with opium, so as to produce salivation, find no favor in his eyes as a remedy for acute rheumatism, and tartar emetic quite as little; as to bark, he shows that it probably disposes to serious complications in the shape of cerebral derangement, and of colchicum given alone he can find nothing favorable to say, except when it excites profuse evacuations from the kidneys, the stomach, or the liver and bowels. Guaiacum he has found serviceable in those cases only which are unaccompanied with perspiration. Nitrate of potash, in spite of the eulogies of Drs. Gendrin, Bennet and others, has not been successful in his hands. Lemon juice he cannot recommend generally. But of the alkalies and their salts he thinks it impossible to speak too highly, although, administered alone, they are quite inadequate to effect a cure. Dr. Fuller employs them in conjunction with all or several of the remedies before mentioned. He says:

"In large but ordinary doses they generally mitigated the severity of symptoms, yet failed in affording more than partial relief, but when they were exhibited in sufficient quantities, and in combination with other remedies, the most agonizing pain was speedily removed, and the fever subdued with marvellous rapidity. I have, therefore, ever since, administered them largely, and have pushed them until my object has been attained. Nor have I seen reason, on any one occasion, to hesitate in following out this plan of treatment. It has now been pursued in a large number of cases, and in almost every instance has produced the most astonishingly favorable results. The patients have speedily lost their pains and have proceeded rapidly to convalescence. In twenty-three out of thirty-nine cases in my note-book, the pulse was tranquillized within forty-eight hours from the commencement of treatment. and in twenty-eight the pain was lulled, and the local inflammation greatly subdued within the same time, whilst in the remaining cases a longer time was required, in consequence either of previous constipation, or of the co-existence of some internal complication.

"The form in which I usually administer the remedies, is that of a simple saline or nitre draught, to which, if the patient be a person of average strength and robustness, bathed in profuse perspiration, with red, swollen, and exquisitely painful joints, a furred tongue, loaded urine, and a full and bounding pulse, I usually add from two to three drachms of the potassio tartrate of soda, ten or fifteen minims of the vinum colchici, from fifteen to twenty minims of the vinum antimonii, and from ten to fifteen minims of the tinctura opii, or of Battley's sedative solution, to prevent the salt running off by the bowels. This draught is repeated, for the first twelve or twenty-four hours, at intervals of three or four hours, according to the strength of the patient and the severity of the attack; and if the pain is excessive, I prescribe a pill containing from half a grain to a grain or a grain and a half of opium, or an equivalent dose of Dover's powder to be taken once or twice a day, taking care to increase or diminish the quantity of the sedative, according to the circumstances of the case; on the one hand avoiding constipation and narcotism, and on the other, guarding against diarrhœa."

Amongst external remedies leeches, blisters, and warm fomentations are recommended. Of the last he prefers alkaline solutions, but, very naturally we should think, found them most successful when mixed with opiates.

In the chapter on rheumatic affections of the heart the author states in opposition to Drs. Latham and Watson, that this complication is most apt to occur when the rheumatic fever is severe. He also found that the subjects of it were weak and pale, reduced by previous illness, or exhausted by the treatment adopted, and wisely learns from this fact a caution against the use of debilitating remedies. He also refutes the prevalent error that the vegetations, &c., sometimes found in the heart afford evidence of endocardial inflammation. The sounds to which they give rise during life are not accompanied by inflammatory symptoms, and true inflammation of the lining membrane of the heart is often seen without such lesions. He regards them as the result of a mechanical action of the valves and their cords, upon the highly fibrinous blood which circulates through the heart. He also thinks that the deposit is sometimes the cause rather than the consequences of valvular inflammation.

The pathological effects of rheumatic inflammation of the heart are very minutely and correctly described, with the general lesions and the physical signs, although the last are stated according to the nomenclature of a theory which takes no account whatever of the auricles as active agents in producing the normal movements and the abnormal sounds of the heart, and there is, therefore, the usual amount of confusion in explaining, according to this theory, the want of correspondence between the præcordial and the arterial pulse.

In the chapter upon the treatment of rheumatic inflammation of the heart the author attacks a prevalent error. It is often said that the use of mercurials in rheumatism tends to ward off cardiac inflammation; the author affirms that they invite this complication, and we fully subscribe to his statement. All that debilitates has the same effect. Nevertheless calomel is the sheet-anchor in the treatment of the attack when once developed; it perfects the impression made by local depletion, which probably has but little influence beyond relieving the pain, or by general bleeding in the few cases in which a high degree of arterial action renders this remedy appropriate. The mercurial must be pushed to the extent of producing ptyalism. When this effect takes place, the local and the general symptoms begin at once to amend. For cachectic and debilitated persons, however, mercury is not appropriate. For these, and indeed for the subjects of the disease generally, opium is an indispensable medicine. But the doses must be large enough to procure quietude and relief from pain. Blisters over the heart are also most valuable remedies when once effusion has taken place.

The proportion of cases in which the heart becomes implicated in the course of acute rheumatism is stated by Dr. Fuller at one half, and although he thinks the number might be greatly reduced by appropriate treatment, yet it would not appear that this influence is one of great weight. For the proportion of cases arising under the phlebotomizing *régime* of M. Bouillaud was 1 in 1.75, and that in the author's own practice 1 in 2.06.

The character of the cerebral symptoms which sometimes complicate this disease is thus delineated :—

"A patient, for instance, who for a week or ten days has been suffering from acute rheumatism, and has presented no untoward symptom, after passing one or two restless nights becomes strange and flighty in his manner, complains, perhaps, of headache, and is shortly seized with furious delirium, during which he appears to be insensible to pain, and moves his limbs in utter disregard of his inflamed and exquisitely painful joints. And then, if he does not shortly improve, he either dies of exhaustion or falls into a state of profound coma, and expires in the course of a few hours."

The most interesting feature of this complication is that the delirium and fatal coma are nearly always quite independent of inflammation of the brain. The author, like Dr. Todd and others, refers them to "the poisoned condition of the blood," but he finds it difficult to explain why they should then occur so seldom. He reverts to the fact that they are generally displayed by feeble, cachectic, susceptible persons, and although he tries, by an analogy drawn from delirium tremens to sustain his view, it seems, after all, to have no other substantiality than belongs to a plausible hypothesis. The treatment found successful is precisely that used in delirium tremens and in traumatic delirium, viz: stimulants and opiates. Cases do sometimes, but very rarely, occur in which true meningitis arises to complicate articular rheumatism, and some also of tetanic spasms, which imply derangement of the spinal marrow.

Inflammation of the lungs and their membranous coverings is another complication which adds greatly to the danger attendant upon rheumatism. This affection has been very well described by our countryman, Dr. T. Buckler, of Baltimore, more minutely indeed than by the author of the work before us, who, however, makes the same general statements regarding the obstinacy of the disease and the efficacy of alkalies in its treatment. This chapter deserves the especial study of the reader, who will probably derive from it new views in regard to the character of certain cases of bronchitis or pneumonia which may have surprised him by their duration, and by the violence of particular symptoms.

Disorganization of the joints is another consequence of articular rheumatism, which has sometimes been called in question, and is certainly of rare occurrence. Some cases recover with permanent stiffness of the affected joint, others terminate fatally, and dissection reveal fibrinous exudation, vascular injection, or purulent deposits within the articulation. The author is of opinion that these disastrous consequences may generally be prevented by warm alkaline fomentations to the affected parts conjoined with perfect rest.

The chapter on rheumatic gout calls for no especial notice, and that on chronic rheumatism contains little that is novel or peculiar. We observe that the author attaches much importance to the use of guaiacum either in the form of tincture or associated with sulphur or cream of tartar. As a remedy for muscular rheumatism hydrochlorate of ammonia he thinks produces "marvellously good results," in fifteen or twenty grain doses, and in combination with bark. Mercurialization he regards as needless, if not hurtful, in ordinary cases, but as sometimes useful when there is excessive tenderness with puffiness about a periosteal swelling, &c., particularly when the constitution is tainted with syphilis. A very gentle course of mercury we should, on the contrary, regard as more likely than any other single remedy to put an end to chronic rheumatism. The concluding chapter is on sciatica and other forms of neuralgic rheumatism. The author appears to take for granted that the pain of the former disease is induced, in many cases at least, by the existence of a fluid within the sheath of the nerve. He does not adduce any proofs of its existence, but seems rather to borrow the idea from Cotugno, whose treatise he refers to as the origin of the revulsive plan of treatment. He does not appear to be aware of the development given to this method within a few years, particularly by M. Valleix, who found the counter-irritant and not the revulsive action of blisters to be the efficient agent in the cure of sciatica, as well as of other forms of neuralgia.

---

*Clinical Lectures on Pulmonary Consumption.* By THEOPHILUS THOMPSON, M. D., F. R. S., Fellow of the Royal College of Physicians, Physician to the Hospital for Consumption and Diseases of the Chest ; Author of Annals of Influenza, prepared for the Sydenham Society, &c., &c. Philadelphia. Lindsay & Blakiston, 1854.

A few months have only elapsed since we had occasion to examine Dr. Bennett's able treatise on Tuberculosis. We have now before us another work devoted to the same affection, in which the pathology of the disease, the principal subject of Dr. B.'s work, is barely noticed. In its place, however, we have presented to us what appeared to the author to be most important to practitioners in the way of direct and practical utility. Consumption is so frequent and so fearful a disease, that numerous as are the writers upon it, we always hail with satisfaction any contributions which promise to throw new light upon either its causes, symptoms, or treatment. Dr. Thompson's position as Physician to the Brompton Hospital for Consumption, affording him rare opportunities for observing and treating the disease in all its different varieties, stages, and connexions, induces us to place much confidence in his opinions. We shall proceed, therefore, to give a short summary of his book, assuring our readers that it will gratify and amply repay a full perusal.

The work consists of thirteen lectures, preceded by a short introduction designed to render auscultation more simple and comprehensible to the student. In the first lecture, the value of the indication, afforded by inspection of the chest, is well shown by the relation of several cases, one of which is the curious instance of hydatids of the liver, which, by absorption and ulceration, found their way into the lungs and were expectorated, provoking cough and causing discharge of purulent matter, night sweats, emaciation and other symptoms of phthisis. The expansion of both sides of the chest being however equal during inspiration and quite free, also, in the sub-clavicular regions, evinced that no serious disease, existed in this instance. The subject of hæmoptysis is taken up in the second lecture. Dr. Thompson asks whether the popular opinion that "breaking a blood-vessel" produces consumption, or the medical one, often implied, if not stated, that hæmoptysis precedes phthisis, is a correct one. As an answer to his query, he gives a table of twenty-four cases, in whom this symptom had occurred. In fourteen of these, other symptoms preceded the bleeding from the lungs; in six, one or other of the symptoms and the spitting of blood commenced at the same time. In four only did there exist no evidence of previously disturbed function. He says:

"You will observe that some of the cases of phthisis recorded in the table, accompanied with copious hæmoptysis, were remarkably slow in their progress. In six of the cases the quantity of blood expectorated at once has exceeded a pint, and the time which has elapsed since the occurrence of the profuse hæmoptysis to the present period has been, in these patients, respectively six months, twenty-two months, twelve months, ten months, eight months, and five years. In several of these instances, evidence of pulmonary disease preceded by many months the occurrence of hæmoptysis, and in some the disease has not yet advanced beyond the first stage. These facts are in harmony with my general experience, as showing that this symptom tends more to retard than to accelerate a fatal issue.

"The practical bearing of these facts is obvious and important, as impressing the conclusion that undue haste to arrest hæmoptysis should be deprecated, and that, as a general rule, it is better to moderate this symptom by producing determination to other organs, than to employ direct astringents. You will find great benefit in many cases from the administration of a dose of calomel or mercurial pill, with henbane, followed by the use of half-drachm doses of sulphate of magnesia with diluted sulphuric acid, administered twice a day."

To arrest hæmoptysis, in urgent cures, the most powerful of direct astringents is acetate of lead. Turpentine is stated to be one of the most certain remedies in a majority of instances.

The third lecture upon expectoration as a means of diagnosis is highly interesting. The characteristic appearance attending the series of changes of the expectoration in the different stages of the disease are described under four divisions: 1st, the salivary or frothy indicating irritation, the result of congestion or of slight tubercular deposit; 2d, the mucous, showing a more confirmed affection of the bronchial tubes; 3d, the flocculent, characteristic of secretion from a vomica, modified by the absorption of its thinner constituents; and 4th, the purulent or porraceous, indicative of phthisis far advanced, and (if unmixed with froth) usually involving both lungs. The 4th lecture is upon the pulse; its acceleration in phthisis, and the effect of posture upon its rate in health, contrasted with the effect in disease, are discussed at great length. Regarding this latter circumstance, his investigations agree with those of Dr. Guy, who has shown conclusively that if the pulse of a healthy person be felt first as he rests with the back supported in an easy chair, and then in the standing posture, a marked difference in frequency is observable, the difference increasing in proportion to the natural frequency of the individual's pulse. The healthy difference is shown in the following table of the rate of increase.

| | | | | |
|---|---|---|---|---|
| Pulse of healthy adult, when sitting, | 60 | 80 | 100 | 120 |
| Ditto, when standing, | 66 | 93 | 119 | 147 |
| Rate of increase, | 6 | 13 | 19 | 27 |

In another table, recording the pulse of the patients in the hospital, it is shown that the difference produced by change from the sitting to the standing position, is, in phthisis, very trifling, especially in the evening.

The fifth lecture takes up the consideration of the remedial effects of cod liver oil. Dr. Thompson states that the records of the hospital afford "an opportunity of comparing the effect of treatment conducted on general principles, irrespective of the use of this remedy, with treatment in which its administration has occupied an important place; and the more carefully you institute the comparison, the more you will be convinced of the value of this medicine in the treatment of phthisis, when appropriately administered, and combined with the use of such other measures as any special circumstances in the individual patient may require."

Several interesting cases are related, showing its efficacy. The following case is, in every respect, so encouraging, that we have concluded to transcribe it.

"M. B., is, I am informed, the only remaining member of a large family, all of whom have died of phthisis. She was admitted five months since, with dull percussion of the right apex; at the left, gurgling in respiration and cough. Her case was examined and recorded by two other medical gentlemen before I explored her chest, and my account corresponded with theirs as to the existence of cavity in the left side. To-day two of my colleagues have examined her, and agree with me in the opinion that no sign of cavity can now be detected in that situation. Let me describe the progress of her improvement; the extent of the gurgling gradually lessened, then dry cavernous respiration was the principal sign; this was superseded by blowing, and then bronchial breathing, and at present I detect nothing wrong except a little flattening of contour, slight dulness on percussion, and wavy inspiration. The catamenia have returned; the pulse has sunk from 112 to 80. Her weight five months since was seven stone twelve pounds and a quarter.* It will be observed that this patient gained twenty-one pounds in weight during the space of twenty-one weeks; and it is worthy of notice, that the quantity of cod-liver oil administered during that time was considerably less than three pints; a fact strongly opposed to the opinion that the oil is useful only in the way of nutritive material. Our usual plan is to give one or two drachms twice a day at first, gradually increasing the quantity to half an ounce three times a day, and I have seldom found any advantage accrue from going beyond this limit."

Dr. Thompson's experience is favorable to the use of liquor potassæ, in combination with the oil, especially in the early stage of phthisis. He believes with Dr. Hughes Bennett, that in scrofulous affections a condition of undue acidity of stomach exists unfavorable to the solution of albuminous materials. "Many patients take the oil unmixed, or, when such combinations are appropriate, floated on nitro-muriatic acid mixture, or in lemonade, or in a saline draught during effervescence. The addition of creasote sometimes renders the stomach more tolerant of the remedy." When the stomach absolutely rebels against it, he uses it endermically.

In the sixth lecture the efficiency of other oils is considered.

* This patient continues, at the present time (August, 1853,) in a state of apparent health.

Dr. Thompson found no remedial effects from the use of almond or olive oils. Of cocoa nut oil he has a high opinion. He says:

"The statement made in this lecture regarding vegetable oils requires an important qualification in consequence of experiments which I have made during the first eight months of the present year (1853) with oil of cocoa-nut, which appears to me to possess medicinal properties similar to those of cod-liver oil. The results in the first thirty patients to whom I administered it bear comparison with those obtained in the first thirty-seven patients for whom I prescribed cod-liver oil, chiefly in the year 1845, as related to the Medical Society of London, and briefly described in some of the medical journals. Amongst the patients to whom cocoa-nut oil was given, there were some instances of arrested phthisis, as decided as any I have been accustomed to attribute to the use of cod-liver oil, over which it possesses advantages in reference to economy and palatableness; and it is interesting to remark that its efficacy was experienced by some who had previously taken cod oil uselessly, and by others who had discontinued it on account of nausea."

We shall pass over the next three lectures, though they contain much interesting matter, that we may have space to notice the subject of the tenth, viz: the appearance of the gums in consumption. Considerable attention to this inquiry has impressed our author with "a conviction of the frequent existence, in consumptive subjects, of a mark at the reflected edge of the gums, usually deeper in color than the adjoining surface, and producing a festooned appearance, by the accuracy with which it corresponds with the curve of the gingival border; this mark is in some patients a mere streak; in others, a margin, sometimes more than a line in breadth. In the most decided cases, this margin is of a vermillion tint, inclining to lake." A plate of this appearance of the gum is given us. The line is usually most distinct around the incisor teeth, though frequently apparent also round the molars. The author asserts that this streak frequently appears among the earliest signs of phthisis, and he regards its absence as favorable.

"As respects the value of this indication in prognosis, I think you will find it a general rule that the early appearance of the streak is an unfavorable circumstance; in cases in which this occurs tending to proceed more rapidly than those in which the streak is absent; whereas freedom from the streak, even in the third stage of the disease, has been particularly noticed in those patients in whom the results of treatment have been most encouraging. Breadth of the discolored margin, and its extension around the molar teeth, you may regard as affording unfavorable indications.

"In reference to diagnosis, there is reason to believe,—

"1st. That the absence of the streak in men affected with inconclusive symptoms of consumption may incline you to a favorable interpretation of any such suspicious indications, but that in women rather less weight is to be attributed to this negative sign.

"2dly. That the presence of the sign in women is almost conclusive evidence of the existence of the tubercular element in the blood.

"When in either sex it coincides with a pulse not materially altered in frequency by change from the sitting to the standing posture, the presence of phthisis may with high probability be assumed, even before having recourse to auscultation.

"The degree in which this appearance exists is not without importance in relation to treatment. When, for instance, the margin is considerable in extent and intensity, it is often advantageous to administer refrigerant remedies, especially salines combined with prussic acid, as a preliminary to the employment of cod-liver oil, or any tonic measures or stimulating diet; and when, as often occurs, diarrhœa accompanies this condition, trisnitrate of bismuth is specially useful. There is reason to believe that the presence of the streak, in some instances, indicates the existence of a tubercular taint in the constitution, before any signs of such a condition can be detected in the lungs. When the streak is absent, whatever be the pectoral symptoms, we have at least one ground for assuming that the constitution is not extensively involved, and we may hope to be able, by the administration of suitable remedies, to promote healthy nutrition, and avert or retard the establishment of phthisis."

The eleventh, twelfth, and thirteenth lectures are upon diseases presenting symptoms resembling those of phthisis, on the causes of consumption and on some of its distressing incidental symptoms. Our readers will perceive the practical nature of the work from the few extracts we have made. In fact, we know of no modern treatise in which, while the physical signs are not neglected, the various manifestations of the disease are so fully discussed. The publishers are entitled to the thanks of its readers for the admirable style in which it is presented to them.

---

*Handbook of Chemistry, Theoretical, Practical, and Technical.* By F. A. ABEL, Professor of Chemistry at the Royal Military Academy, Woolwich, and C. L. BLOXAM, formerly First Assistant to the Royal College of Chemistry. *With a preface by* DR. HOFFMAN. Philadelphia: Blanchard & Lea, 1854.

On opening the work before us, we are reminded of a fault of more frequent occurrence in treatises on Chemistry, than on any other branch of science. We allude to the want of a clear conception on the part of the author of the object or purpose to be fulfilled by his publication. The difficulty is not what should be *inserted*, but what can be *omitted*. The subject matter of the science is so unlimited, and the uses to which it is applied so manifold, that it is impossible for one volume, or indeed one treatise, to be at the same time a text book for the medical student, a hand-book for the analytical chemist, and a dictionary of arts for the practical man and general reader. On taking up a work, therefore, which claims for itself higher rank than a mere sketch of the science (which must necessarily be superficial) we cannot but be disappointed when we find several branches of Chemistry included in one volume, to do justice to which a dozen would scarcely be sufficient. The title of the work under question "The Hand-book of Chemistry" led us to suppose it was a treatise on the science of chemistry generally, organic and inorganic. We were therefore surprised to find the former entirely omitted, the more so, since the authors say very properly in their introduction, "The science of chemistry is usually divided into two branches, *inorganic* and organic chemistry; as a convenient mode of classifying our knowledge this division is useful, but as a natural and absolute separation it has no existence; for the two classes of substances, inorganic and organic, so verge into each other, so many so-called organic substances are found capable of being prepared by inorganic methods, that the boundary line is day by day becoming fainter and will perhaps in time vanish altogether." The title of the work should, therefore, be "The Hand-book of Inorganic Chemistry."

The book opens with the usual definitions of the science. Specific gravity and the means used for its determinations; the explanation of chemical terms; equivalents, affinity, nomenclature, and notation are next given. A chapter on crystallization and crystallography closes this, the introductory portion of the work. Instructions in chemical manipulation, the arrangement of apparatus, collection of gases, management and application of heat come next, occupying about one-eighth of the book, very useful as far as they go, though, from the limited space, of course not equal to Farraday's manipulations, or to Moffat's book on the same subject. The usual systematic course of the elements and their inorganic compounds then commences, and contains all the new discoveries and additions of note, the whole subject being well arranged and treated more at length and in detail than is customary in the English Chemistries. Interspersed through this part are the outlines of the practical manufacture of sundry articles; for instance, glass, gunpowder, iron, etc., which, we think, might have been omitted without injury, as they take up in the aggregate considerable room, and although of some general interest, are out of place and do not add to the real value of the book. The remainder of the book, about one fourth of the whole, is devoted to instruction in qualitative and quantitative analysis, with directions for experimental analysis for the practice of the student, preceded by descriptions of the necessary apparatus, etc. The analysis of soils, ores, mineral waters, etc. closes the volume.

It is to this latter part, *i. e.* analytical chemistry, that the value of the work is due, and to which the rest of the book is subservient. It is evident that the authors are practically familiar with the subject, and have given their personal experience to guide the student. The choice of subjects for examination, the lists of re-agents, and directions for the detection of the various substances as suspected, are clear and satisfactory, and render the book a useful guide and assistant to the student in analysis.

The book is thus divided into three independent treatises, viz.: first, Chemical Manipulation; second, Systematic description of the elements and their inorganic compounds; and lastly, Analytical Chemistry. These would seem to be quite enough to group together into one volume, without also adding manufacturing processes, that can be but of little use to the analyst, for whose benefit the work is intended. The theory of the operations would have been sufficient, at least, in the present case. As a necessary consequence, something must be omitted to keep the book within reasonable limits, and the chemistry of vegetable and animal life, and of the compounds described therefrom, has been chosen as a subject readily dispensed with, without hurting the general plan of the work, and is therefore left out, of course much to the detriment of its practical value, particularly to the physician. Directions for the detection of a few organic substances are given; for instance, the tartrates, citrates, urates and their respective acids, and a few other compounds, but too limited to be of much value.

We notice a few errors that a little care might have avoided; for instance, the composition of tartrate of lime is given ($2CaO\bar{T}$) instead of ($2CaO\bar{T}+8Aq.$) As tartrate of lime is generally used for the quantitative determination of tartaric acid, the error is important. Again, in describing the manufacture of pot and pearl ash, the process of making pot-ash is confounded with that for pearl ash, and pearl ash is mentioned as the product of a process, which is really that for obtaining salt of tartar.

The American edition has the advantage over the English one, in being illustrated with numerous wood cuts, showing the construction and form of apparatus, the shape of crystals, etc., the cuts being mostly taken from the American editions of Fowne's and of Regnault's Chemistries. The English edition was without illustrations. The print and paper are good, and the work neatly bound; it forms on the whole, a useful addition to the library of the experimental student.

---

### TRANSACTIONS OF THE AMERICAN MEDICAL ASSOCIATION.

(Concluded.)

*On Acute and Chronic Diseases of the Neck of the Uterus.* By CHAS. D. MEIGS, of Philadelphia.

This report, comprising 51 pages, illustrated by several colored engravings, was not intended by the author to present a complete summary of what has been written upon the subject, but was prepared merely to serve as a useful aid to clinical practice in an extensive department of disease. We need hardly inform the reader, that Dr. Meigs' treatment of the subject exhibits his accustomed originality, nor that his style is dainty and unique, as is its usual characteristic. After stating that the re-actions of the re-productive organs upon the other members of the human economy are so many and great that "changes in their vital status, even such as are inappreciable, except by the understanding, greatly affect both the mind and body of the woman," he says, "a dark and mysterious veil hides from us many of the laws that grow out of the intimate relation and mutual dependence existing between the conservative or genetic forces of animals, and their reproductive or genetic powers." "Many stumbling-blocks in the path of the practitioner would be taken away, if these laws and relations could be fully understood; and we should then be able to take more precise indications, and adopt more positive methods of treatment. Possessing full anatomical and physiological information upon those organs and forces, we might raise up in the mind a true *ideal* of them, which would serve as a standard or scale by which to measure and judge every aberration of form, power or place, in the instances brought before us."

"A perfect ideal of the normal womb, one fit to serve as a standard, or scale of comparison or measurement for cases, should comprise, in addition to a notion of its elements, one of its form, volume, place, posture in that place, sensibility, resistance, complexion and all its powers, as well as its anatomical relations or connection." As a help to the formation of this ideal standard, several figures copied from nature with explanations, are given us of the womb and its relative position in the pelvis.

One of the most common of sexual disorders is leucorrhœa. Most women suffer from this affection at some period of their lives. In general, it is not profuse and ceases spontaneously. When the discharge consists only of vaginal products it is of little consequence. It is hurtful only when it comes from the canal of the cervix. The discharge differs according to its seat. "The muciparous glands of the vagina furnish either a thin watery mucus, or one of a creamy consistence, which, in other instances, is almost butyraceous or concrete.

The excretion from the follicles and glands of the canal of the neck is always gluey or albuminous, and resembles fresh white of eggs; and when the patient, in describing the disorder, informs us that she discovers a slimy mucus, and especially if it appears at intervals of once a day or oftener, we may take it for granted that she labors under inflammation of the neck of the womb."

The lassitude and debility accompanying leucorrhœa is not the effect of the excessive secretion, but is due to "the disturbing effect in the general economy, produced by even slight modifications of the health of the uterus." Serious cases are cases of disease of the cervix, and are to be treated accordingly. For the diagnosis of the affection, the touch, "where it gives sufficient information, may suffice; if there be any doubt, the speculum must be used. The most reliable metroscope, probably, is a slightly conical tube of silver, six inches and a half in length. The uterine extremity should be one inch in diameter, bevelled with an angle of thirty-five or forty degrees; the outer or larger extremity should be one inch and a half in diameter. The silver should be highly polished, with the edges of the bevelled end rôlled and rendered blunt, lest they might catch in the folds of the membrane, or even wound the cervix.

An olive shaped piece of wood, secured in a steel handle, and made to fit accurately in the smaller extremity of the cone, serves to conduct it without pain to the bottom of the vagina, whereupon it is withdrawn, in order that the surgical neck may engage in the opening, and thus enable the surgeon to discern any marks of disease there.

The light passing down the tube ought to be as clear as possible, and the inner surface of the metroscope ought not to have a very high polish, lest serving as a reflector, it should pour a flood of chromatized light on the parts, and thus give rise to the greatest misapprehension of their real condition. It would be better to have the inner surface painted with black, in order that no reflection from the walls should deceive us, and lead to error."

"There should be provided a speculum forceps, and some small bits of moistened sponge, which being held in the forceps, serve to absorb or remove any mucus or slime or sanguineous excretion. The most convenient one is a "bullet forceps." If an examination be now made to determine the cause of the leucorrhœa, we shall rarely fail to observe a positive inflammation of the cervix and os uteri, or to notice a plug of viscid phlegm in or issuing from the os. One or both lips of the womb will be found tumid, softened, granulated, and of a uniform red. Plates exhibiting these appearances (inflammation of the cervix and partial and general hypertrophy of the womb) are given, and the cases described in which they occurred.

The remedy advised is "antiphlogistic contacts of the nitrate of silver." To apply these properly, the practitioner "must create for himself an *ideal* of his operation, so that when about to perform it, he may predetermine what it is he hath to do, and whether the contact he is going to make shall be a destructive or an antiphlogistic one.

"The absence of precision in the design and act, frequently occasions the greatest and most poignant suffering, which, in our estimation, is wicked and abominable; they are disgraceful to the art and the artist at once."

Dr. Meigs' large experience has satisfied him that ulceration of the womb is among the rarest of disorders. The framboisée inflammation and hypertrophies have been taken for ulcerations, the superficies being covered with a delicate epithelium, very liable to give way under unskilful manipulation.

"A proper antiphlogistic and resolvent contact of the crayon ought not to destroy even this delicate epithelium; but rather to make it more firm and dense, and so planish, as it were, the unevenness down to the normal surface level. In this way we may compel the densy or tubercular eminences to sink down again to their place, and give, by solidifying the epithelium, a firm physical delimitary support to the before debilitated capillaries that rise up in the form of a soft molluscum."

As another consequence of uterine irritation and hypertrophy, retroversion frequently takes place. Dr. Meigs is convinced that it constitutes seventy-five per cent. of all cases of sexual disorder, of a gravity sufficient to require appeal to medical advice. Retention of urine, a common habit with women, is the most frequent cause of this affection.

Several interesting cases of retroversion and of small bleeding tumors attached to the os by a delicate peduncle, accompanied with hypertrophy of the womb, are here detailed. Of the treatment of retroversion, he observes:

"An immense use has been made of the globe pessary; and certainly, in a simple prolapsus uteri, it answers admirably. But it does not answer well for the cases of retroversion." For these Dr. Meigs recommends a circular pessary, made of watch spring, wound round with bobbin. This is to be dipped in melted virgin wax and then polished. Being elastic it can easily be introduced by compressing the sides. Properly lodged, according to Dr. Meigs, it renders retroversion impossible.

We have given a very slight sketch of Dr. Meigs' report. It is well worthy of study by the profession, containing, as it does, much valuable information on the subject of which it treats, written in a lively and agreeable style.

We here conclude our notice of the transactions. The volume contains a number of other reports worthy the attention of our readers, but for which we must refer them to the book, our limits forbidding further mention.

---

### MEDICAL SOCIETY OF THE STATE OF PENNSYLVANIA.

The Medical Society of the State of Pennsylvania, met on Wednesday, May 31st, 1854, at the Court House in the City of Pottsville, and, in the absence of the President, was called to order by the Vice President, Dr. CONFER, at 11 o'clock, A. M.

The following were appointed a Committee, to examine and report on the credentials of Delegates:—JOHN NEILL, Philadelphia Co.; C. W. PARRISH, Chester Co.: WM. MAYBURRY, Philadelphia Co.

On motion of Dr. WEST, Dr. D. FRANCIS CONDIE, of Philadelphia County, was appointed Recording Secretary, pro tempore, to supply the vacancy occasioned by the death of Dr. Henry S. Patterson.

The following delegates answered to their names.

*Berks County*—C. H. Hunter, E. Kitchen, W. T. Bladen, Martin Luther, J. H. Spatz.

*Bradford*—Geo. F. Horter.

*Blair*—J. M. Confer.

*Bucks*—Jno. Dyer, Jr.

*Chester*—C. W. Parrish, S. A. Ogier, W. W. Townsend, Chas. E. Coates, A. K. Gaston, J. R. Walker.

*Delaware*—R. K. Smith.

*Huntingdon*—H. K. Neff.

*Lancaster*—H. Carpenter, P. Cassady.

*Lebanon*—B. F. Schneck, W. Moore Guilford.

*Lehigh*—Chas. E. Hoffman, David O. Mosser.

*Mifflin*—T. Mitchell, E. W. Hale.

*Montgomery*—Jno. A. Martin, Winthrop Sargent, Jno. Schrack, Hiram Corson, Lewis W. Read, Wm. A. Van Buskirk.

*Northampton*—R. E. James, Charles Innes.

*Perry*—J. H. Case, James Galbraith.

*Philadelphia*—T. E. Beesley, T. F. Betton, D. Francis Condie, J. M. Greene, H. Hartshorne, S. L. Hollingsworth, Jacob Huckel, B. S Janney, Wilson Jewell, A. L. Kennedy, Samuel Lewis, W. Mayburry, John Neill, J. M. Pugh, W. S. W. Ruschenberger, M. B. Smith, R. P. Thomas, Francis West, Geo. B. Wood.

*Schuylkill Co.*—A. Heger, Wm. Housel, J. W. Gibbs, J. F. Frechte, J. G. Kochler, J. H. Wythes.

Total present 59.

The Committee on Credentials reported, that there was no delegate accredited from Huntingdom county Medical Society. Dr. H. K. Neff, of that county, and an Associate of this Society, was, however, in attendance, and the Committee recommended that he be received and recognized as a delegate. Upon motion of Dr. Neill the said recommendation was approved.

On motion of Dr. Pugh, the reading of the minutes of the last annual session was dispensed with, and the same were, on motion of Dr. Thomas, referred to a committee, consisting of Drs. Thomas and Ruschenberger, of Philadelphia, and Carpenter, of Lancaster, to report any unfinished business that may appear therein.

The Committee of Publication presented their report, as follows:

The Committee of Publication beg leave to report that they have had printed seven hundred and fifty copies of the Transactions of the State Society, at its session of 1853. The expense of which was $262 15.

The copies were distributed to the several County Societies represented, in proportion, as nearly as could be done, to the number of their members respectively.

It would be a desirable thing, could it be accomplished, to place at the disposal of the Committee of Publication a fund, that would enable them to print a sufficiently large edition of volumes of Transactions, to supply a copy to each member of the several societies represented at the sessions of the State Society.

Signed, on behalf of the Committee, by

Dr. D. Francis Condie, Chairman.

The report was received and ordered to be inserted on the minutes.

The Treasurer, Dr. West, presented his report, which was referred to an auditing committee consisting of Drs. C. W. Parrish, W. A. Van Buskirk, and E. W. Hale.

The Committee on Unfinished Business presented the following report:

The Committee appointed to present from the minutes of the last session such items of unfinished business as may appear thereon, respectfully report, that they find reports to be due from the following committees, namely:

1. A Committee, consisting of Drs. Biddle, Parry and Carson, appointed to report on the resolutions of the Berks County Medical Society, relative to the collection of samples of drugs and preparations (see printed Minutes of 1853, page 12.)

2. A Committee on gratuitous vaccination throughout the State, Dr. G. Emerson, Chairman, appointed in 1852, and continued in 1853, with direction to report at the present session, (see printed Minutes, 1853, pp. 15, 19.)

3. A Committee, consisting of Drs. Parry, Cassady and Carpenter, on vaccination and the preservation of kine-pock, (see printed Minutes of 1853, page 21 )

Signed, R. P. Thomas,
W. S. W. Ruschenberger.
Henry Carpenter.

Dr. HOLLINGSWORTH, on behalf of the Chairman on samples of drugs, etc., tendered an apology for the non-presentation of the report, when, on motion, the Committee was continued, with instructions to report at the next session.

Dr. CARPENTER, on behalf of the Committee on vaccination and the preservation of vaccine matter, stated that the committee had not been able to prepare, in time for presentation at the present session, all the materials for their report, but hoped to be able to have it completed by the next session.

The Committee on the Gratuitous Vaccination of the Poor, presented the following report:

The Committee appointed to memorialize the Legislature of the State for the passage of an act to provide for the gratuitous vaccination of the poor throughout this Commonwealth, report:

That, since their previous report, having given to the subject entrusted to them more mature consideration, they are persuaded that the main object originally contemplated by this Society, namely, the universal extension of the protection afforded by vaccination to the entire population of our State, may be more effectually secured through the efforts of the authorities entrusted with the management of public affairs in the several counties, than by officers appointed by the State government. The duty of providing for the poor is entrusted to the respective counties, as well as the direction and control of the public schools. The committee feel persuaded that, by the combined action of the county officers and the directors of the public schools, with the powers already delegated to them, much may be effected towards promoting a more general extension of the protection afforded by vaccination throughout the State. To carry out these views, they therefore offer the following resolution:

Resolved, That in order to extend the benefits of vaccination as far as possible among the inhabitants of this Commonwealth, the State Medical Society urge upon the several organized County Societies throughout the State, to use their influence to induce the public authorities of their respective counties to adopt prompt measures for as general an extension of vaccination as may be practicable; and to render it imperative upon those who are charged with the direction or superintendence of schools, public as well as private, to require, before receiving any pupil, a written certificate from some properly authorized physician, that he or she has undergone successful vaccination.

Signed, G. EMERSON,
D. FRANCIS CONDIE.
WILSON JEWELL.

On motion of Dr. CARPENTER, the report was received, and the resolution appended thereunto was unanimously adopted. On motion of Dr. BETTON, it was resolved, that the said report be printed, and a copy thereof sent to each of the County Societies now organized in the State, and where no society is organized, to the Superintendent of the Public Schools of such county.

The Secretary, Dr. I. R. WALKER, reported that he had fulfilled the duty enjoined upon him by the resolution of last session, recorded on page 16 of the printed Minutes.

On motion of Dr. HOLLINGSWORTH, it was resolved, That a committee of one from each county delegation in attendance, to be selected by the respective delegations, be appointed to nominate officers for the ensuing year.

On motion of Dr. CONDIE, it was resolved, That the appointment of of said committee be postponed until to morrow morning.

On motion of Dr. JEWELL, it was resolved, That the said committee be empowered also, to select a place for the meeting of the Society in 1855.

The Committee appointed to audit the accounts of the Treasurer, made the following report:

The committee appointed to audit the account of the Treasurer have performed that duty, and report that, at the time the account was made up by that officer there was a balance in his hands due the Society of thirty-three dollars; since the meeting of the present session he has received the assessments due from Lebanon, Mifflin and Bradford County Medical Societies, leaving now in his hands a balance due the Society of sixty-two dollars. Assessments are still due from Erie, Lycoming, Mercer and York County Medical Societies.

Signed, C. W. PARRISH,
W. A. VAN BUSKIRK,
E. W. HALE.

On motion of Dr. THOMAS, it was resolved, That the meetings of this session shall be from 9 to 12 o'clock in the morning, and from 4 to 6 o'clock in the afternoon.

On motion, resolved, That the President be requested to deliver his address this afternoon, immediately after the reading of the Minutes.

*Afternoon Session, May 31st,* 1854.

The minutes of the morning session were read and approved.

The President, Dr. JOHN P. HIESTER, read the Annual Address.

On motion of Dr. JEWELL, it was resolved, That the thanks of this Society be presented to Dr. HIESTER for his very able address, and that a copy of the same be requested for publication in the Transactions of the Society, it being understood that Dr. Hiester have permission to make, first, such alterations in said address as he may deem necessary and proper.

The Report of the Berks and Blair County Medical Societies were presented and read, and, on motion, referred to the Committee of Publication.

*June 1st,* 1854.

The minutes of yesterday afternoon session were read and approved.

On motion of Dr. KENNEDY, a Committee (Drs. A. L. KENNEDY, D. F. CONDIE, S. L. HOLLINGSWORTH) was appointed to prepare and submit to the next annual session of this Society, forms of printed blanks for County reports. Said blanks to be so worded as to facilitate the preparations of the reports, and to secure greater fulness and uniformity therein.

The following were announced as constituting the Committee of Nomination :—

W. T. Bladen, Berks ; R. K. Smith, Delaware ; E. W. Hale, Mifflin ; G. F. Horter, Bradford ; H. K. Neff, Huntingdon ; J. A. Martin, Montgomery ; J. M. Confer, Blair ; H. Carpenter, Lancaster ; C. Innes, Northampton ; J. Dyer, Jr., Bucks ; B. F. Schneck, Lebanon ; J. H. Case, Perry ; W. W. Townsend, Chester ; D. O. Mosser, Lehigh ; S. L. Hollingsworth, Philada. ; J. G. Koehler, Schuylkill.

On motion of Dr. CASSADY, a Committee (consisting of Drs. M. B. SMITH, H. CARPENTER and WM. MAYBURRY) on obituary notices of deceased members of County Societies, was appointed, with instructions to report this afternoon.

Dr. BETTON offered the following amendment to the Constitution, Art. III., Sec. 2, to insert after the word "societies," in the second line, the words "except in the case of the Philadelphia County Medical Society, which shall hereafter be recognized as the Philadelphia City Medical Society."

Various other amendments to meet the case of the Philadelphia County Society, under the change in the political organization of Philadelphia County, were suggested, when, on motion of Dr. JEWELL, the whole subject was referred to the Philadelphia delegation, with instructions to report at the afternoon session.

The following resolutions were presented by Dr. WYTHES, and, on motion of Dr. CONDIE, referred to the Committee on Forms for County Reports.

*Resolved*, 1st. That the County Societies are recommended to appoint their annual committees to prepare reports as early as practicable after the meeting of the State Society ; and that said committees be sub-divided into sections having reference to the different topics of medical information, viz., Pathology and Therapeutics, Surgery, Midwifery.

2d. Papers read before County Societies should be referred to the appropriate section of the committee, to be used by it in the preparation of the annual report. Papers of more than ordinary merit or interest, to be reported at length, or an abstract made of them at the option of the society.

3d. It is desirable that the reports from the County Societies should furnish, in addition to the general information referred to above, as much statistical intelligence as possible with reference to their respective topics, and the localities of their societies.

The reports from the Bradford, Chester, Delaware, Huntingdon, Lebanon, Lehigh, Mifflin and Montgomery County Medical Societies were presented and read, and, on motion, referred to the Committee of Publication.

*Afternoon Session, June 1st,* 1854.

The minutes of the morning session were read and approved.

The Philadelphia County delegation, to whom the subject was referred, presented the following Preamble and Resolution:

*Whereas,* The recent action of the Legislature of Pennsylvania has consolidated the City and districts of Philadelphia under one municipal government, therefore

*Resolved,* That should the Philadelphia County Medical Society consider it expedient to re-organize, and to change their title in correspondence with the new political organization of the County, it shall have, when thus reorganized, all the rights and privileges of the present Philadelphia County Society; and that the Constitution of this State Society be amended, by inserting in the second line of Sec. 2, Art. III., the words "and from the Philadelphia City Medical Society."

On motion of Dr. CONDIE, the said resolution, including the amendment to the Constitution, was unanimously adopted.

The Committee on Nomination of Officers reported as follows:

*President*—JACOB M. GEMMILL, of Huntingdon Co.

*Vice Presidents*—WILSON JEWELL, Philadelphia Co.; E. W. HALE, Mifflin Co.; WM. HOUSEL, Schuylkill Co.; A. K. GASTON, Chester Co.

*Recording Secretaries*—D. FRANCIS CONDIE, Philadelphia Co.; H. CARPENTER, Lancaster Co.

*Corresponding Secretary*—JOHN NEILL, Philadelphia Co.

*Censors, 1st and 2d Districts*—J. B. Biddle, Philadelphia; J. T. Huddleson, Delaware; Hiram Corson, Montgomery; G. F. Horton, Bradford; P. Cassady, Lancaster; Wm. Griess, Berks; Chas. H. Martin, Lehigh; R. E. James, Northampton.

*3d and 4th Districts*—Thos. Wood, Lycoming; J. B. Luden, Huntingdon; Jos. Henderson, Mifflin; W. W. McIlvaine, York; J. H. Case, Perry.

*5th and 6th Districts*—J. P. Gazzam, Alleghany; W. Anderson, Alleghany; Jas. Dickson, Alleghany; C. F. Perkins, Erie; J. T. Ray, Mercer.

*Delegates to the American Medical Association*—J. P. Hiester, Berks; W. W. Townsend, Chester; Chas. Innes, Northampton; John G. Koehler, Schuylkill; J. H. Dorsey, Huntingdon; Henry Carpenter, Lancaster; John A. Martin, Montgomery; David Mosser, Lehigh; Jas. Galbraith, Perry; Jacob M. Confer, Blair; R. K. Smith, Delaware; Joseph Henderson, Mifflin.

The Committee also reported that they had selected Hollidaysburg as the place of meeting of the Society in 1855.

On motion of Dr. CONDIE, the Committee was continued, with instructions to nominate the additional members of the Publication Committee.

The reports of the Perry County Medical Society, Philadelphia County Medical Society and Schuylkill County Medical Society were presented and read, and, on motion, referred to the Committee of Publication.

The Committee of Nomination reported that they had selected the following gentlemen to compose, with the Recording Secretaries and Treasurer, the Committee of Publication:

FRANCIS G. SMITH, Philadelphia; SAMUEL LEWIS, Philadelphia; ROBERT P. THOMAS, Philadelphia.

The Committee on Obituary Notices presented the following report;

The Committee on obituary notices of deceased members of County Societies beg leave to report, that they have given to the subject all the attention which the short time allotted to them for its consideration would admit, and have come to the conclusion that the object contemplated would be most effectually obtained by the several County Societies presenting in their annual reports a short notice of such members of their societies respectively, as have died during the year. The Committee therefore recommend the adoption of the following resolution:

*Resolved*, That the several County Medical Societies be earnestly solicited to append to their annual reports brief notices of such of their members as may have died during the year.

Signed, MOSES B. SMITH,
WM. MAYBURRY,
HENRY CARPENTER.

The report was accepted, and the resolution adopted.

Dr. PARRISH, on behalf of the Chester County Medical Society, presented the following questions:

1st. Does dysentery prevail most in those in those sections of country in which there is little or no iron in the soil?

2d. Do paralysis and other nervous affections prevail most in those sections of country in which iron is diffused throughout the soil?

3d. Does the clearing off of timber tend to increase affections both of the nerves and of the intestines? and, if so why?

On motion of Dr. OGIER, the said questions were referred to a special Committee, with Dr. A. K. Gaston as Chairman. In addition to whom the President appointed Drs. W. M. Guilford of Lebanon, A. Heger of Schuylkill, L. E. Kitchen of Berks, and Chas. E. Hoffman of Lehigh.

Dr. CORSON presented the following resolution, which, on motion of Dr. WYTHES, was referred to the Committee on forms for County reports:

*Resolved*, That it should always be distinctly stated in those County reports, in which reference is made to the influence of limestone formations in the production or modification of diseases, whether the limestone lies near the surface or at a great depth, and if the latter is the case, what covers it. Also, whether the water used for drinking and cooking is impregnated with calcareous matter.

Dr. R. K. SMITH presented the following preamble and resolutions, which were adopted:

*Whereas*, At the session of the State Medical Society in 1852, a preamble and resolutions, offered by Dr. Emerson, were adopted, urging upon the members of the Medical profession the importance of our present County organization; which preamble and resolutions were re-adopted at the session of 1853, and whereas, the duty therein enjoined upon the President and Secretary of this Society does not appear to have been fulfilled. Therefore

*Resolved*, That the aforesaid preamble and resolutions be again sanctioned by this Society, and the Secretary be requested to communicate a copy of the same to each County Society in the State, and in the Counties in which societies are not organized, to such members of the profession as he may deem proper.

*Resolved*, That the Committee of Publication be authorized to assess upon the County a sum sufficient to carry the foregoing resolution into effect.

Dr. CARPENTER, of Lancaster, stated, that the copy of Transactions ordered to be sent to the "London Epidemiological Society," was transmitted by Dr. ATLEE; the Society have acknowledged its receipt, and send in return a vote of thanks, accompanied with a copy of a report "on the state of small pox and vaccination in England, Wales and other countries, and on compulsory vaccination," a report comprising the most interesting features of which, will be made to this Society at a future period.

Dr. BLADEN, of Berks, presented the following resolution, which, on motion of Dr. THOMAS, was laid on the table.

*Resolved*, That this Society most cordially approves of the resolutions adopted by the Medical Society of the City of Reading and County of Berks, in regard to individuals whose names are found on the "Black List" of Physicians; and that it is hereby recommended to all the County Societies within the State to adopt a similar measure.

A list of the officers and members of the Lancaster County Medical Society was presented and ordered on file.

On motion of Dr. WALKER, it was Resolved, That the Constitution and By-Laws of this Society, and the Code of Ethics, be appended to the next volume of Transactions.

On motion of Dr. WEST, it was Resolved, That the Committee of Publication be instructed to prefix, hereafter, to each annual volume of the Transactions of this Society, a statement to the effect that the Medical Society of the State of Pennsylvania does not hold itself responsible for the facts and opinions contained in any address or report which may be made to it and ordered for publication in its Transactions.

On motion of Dr. THOMAS, the resolution offered by Dr. Emerson at the last session of this Society and indefinitely postponed, (see page 16 printed Minutes,) was taken up and passed.

Dr. MAYBURRY presented the following preamble and resolutions, which were unanimously adopted:

*Whereas*, It has pleased our Creator, in his infinite wisdom, to remove from this temporary sphere of probation to his eternal home, Dr. HENRY S. PATTERSON, our late efficient *Secretary* and highly gifted colleague; therefore *Resolved*, That this Society has received with unfeigned regret the intelligence of the death of Dr. Patterson in the prime of life and usefulness, and that, in common with the Medical profession of this State, we deeply deplore the irreparable loss which, by this afflictive dispensation, the interests of medical science and of medical organization have sustained.

*Resolved,* That a copy of the preceding preamble and resolution be transmitted to the family of the deceased, with the condolence of this Society on their sad bereavement.

Dr. HENRY CARPENTER presented the following preamble and resolutions, which were unanimously adopted.

*Whereas,* It has been the will of an All-Wise Providence to remove by death from among us our much esteemed and deeply lamented friend and colleague, Dr. FRANCIS S. BURROWES, of Lancaster, a most zealous and useful member, and one of the Vice Presidents of this Society, it becomes the melancholy duty of his surviving associates to express their sincere sorrow for the loss they have sustained, of one so distinguished for his ability as a physician, his high character for virtue and integrity as a man, and his many endearing qualities as a companion and a friend. Therefore, *Resolved,* That this Society has heard with deep regret of the decease of their late associate and professional brother; and, while they will ever hold in lively remembrance the recollection of his many virtues, they offer to his family and friends their heartfelt condolence and sympathy.

*Resolved,* That the Recording Secretaries transmit to the family of the deceased a copy of the foregoing.

Dr. MOSES B. SMITH presented the following preamble and resolutions, which were unanimously adopted.

*Whereas,* It has pleased our Heavenly Father, in his inscrutable wisdom to remove from works to rewards, since our last annual meeting, another of the convention that organized this Society, DR. JOSEPH D. STEWART, of the City of Philadelphia, and for some time the resident physician of the Philadelphia Hospital, Blockley; therefore, *Resolved,* That, in the death of Dr. Stewart, this Society and the community at large have lost one of the most useful, amiable, and upright of their members, and that, in this afflictive dispensation of Divine Providence, we heartily sympathize with the bereaved and mourning family of the deceased. *Resolved,* That a copy of this minute be forwarded to the family of the deceased, as a testimony of the high estimation in which he was held by us.

On motion of Dr. R. K. SMITH, it was unanimously resolved, that the officers reported by the Committee of Nomination, be and they are hereby declared to be the officers of this Society for the ensuing year.

On motion of Dr. CARPENTER, the thanks of the Society were tendered to Dr. Hiester, its President, and to the other officers for the able and courteous manner in which they have performed their respective duties.

On motion of Dr. CONDIE, the thanks of the Society were presented to the Commissioners of Schuylkill County for the use of the very beautiful and commodious room in the Court House at Pottsville, for its sessions of the present year.

On motion of Dr. KENNEDY, it was Resolved, that the thanks of this Society be tendered to the Medical profession of Pottsville, for the cordial and hospitable manner in which they provided for the accommodation of the Sessions of the Society, and the attentions bestowed by them upon its members.

On motion of Dr. CARPENTER, it was Resolved, that the next meeting of the State Society be held on the last Wednesday of May, 1855, at 10 o'clock, A. M., in Hollidaysburg.

Adjourned.

